# Whole genome sequences of four potential indigenous probiotics strains isolated from sediment of Sundarbans mangrove and wild black tiger shrimp (*Penaeus monodon*) from the Bay of Bengal, Bangladesh

**DOI:** 10.1128/mra.00792-26

**Published:** 2026-10-23

**Authors:** Shawon Ahmmed, Tahara Rohomania, Mohammad Shamsur Rahman

**Affiliations:** 1 Aquatic Animal Health Group, Aquaculture Program, Department of Fisheries, Faculty of Biological Sciences, University of Dhaka 95324, Dhaka, Bangladesh; Montana State University, Bozeman, Montana, USA

**Keywords:** Indigenous probiotics, Bangladesh Tiger Shrimp, *Bacillus *spp., *Photobacterium ganghwense*, Illumina MiSeq sequencing

## Abstract

Here, we report the whole genome sequences of four potential indigenous probiotic strains: *Photobacterium ganghwense* MSR_IP16, *Bacillus subtilis* MSR_IP31, and two *Bacillus velezensis* (MSR_IP55 and MSR_IP80) isolated from sediment of Sundarbans mangrove and brood shrimps from the Bay of Bengal, Cox’s Bazar, Bangladesh. Genome assemblies ranged from 3.9 to 5.3 Mb with 19–44 contigs, respectively.

## ANNOUNCEMENT

Probiotics are widely used in shrimp aquaculture as sustainable alternatives to antibiotics due to their ability to inhibit pathogens, promote growth, and stimulate host immune responses (1–3). In this study, *Photobacterium ganghwense* MSR_IP16 and *Bacillus subtilis* MSR_IP31 were isolated from sediment of Sundarbans mangrove in Khulna, Bangladesh, and *Bacillus velezensis* MSR_IP55 and MSR_IP80 strains were isolated from black tiger shrimp collected from the Bay of Bengal, Cox’s Bazar, Bangladesh.

The samples were processed and cultured on Bacillus Agar (HiMedia, USA) plate at 37°C for 24 h to isolate potential probiotics strains, which were purified by repeated streaking. The strains were subsequently characterized by a series of biochemical tests (4, 5) followed by *in vitro* antagonistic activity (6), safety, and tolerance assays (7–9). To isolate high-quality genomic DNA, a single colony of these four strains was cultured on TSA plate at 37°C for 24 h. Genomic DNA was extracted using Wizard Genomic DNA Purification Kit (Promega, USA), and libraries were prepared with Nextera XT DNA Library Preparation Kit (Illumina, USA) and employed for 150 bp paired-end whole-genome sequencing using Illumina MiSeq platform (Illumina, USA). Adapter and low-quality reads were trimmed using Trim-galore v0.6.10 (10), and quality was assessed with FastQC v0.12.1 (11). Genomes were assembled with SPAdes v4.2.0 (12) and polished using Pilon v1.24 (13). Genome completeness and contamination were determined by CheckM v1.2.5 (14). Quality and coverage of assembled genome were evaluated using Quast v5.3.0 (15) and Qualimap v2.3 (16). Taxonomic identification and average nucleotide identity (ANI) were determined using AutoMLST (https://automlst.ziemertlab.com/) (17). Genome annotation was performed with Bakta v1.3.0 (18) and Prokka v1.12 (19), while metabolic features, CRISPR arrays, antimicrobial resistance genes, and prophages were identified using RAST server (https://rast.nmpdr.org/rast.cgi) (20), CRISPR/CasFinder v1.1.0 (21), ResFinder v4.7.2 (22), CARD RGI v1.4.2 (23), and PHASTEST v1.0.2 (24), respectively. Virulence-associated genes and presence of plasmids were detected using VFanalyzer v6.0 (25) and PlasmidFinder v3.0.3 (26). The circular genome maps were generated using Proksee (Build: 22 June 2026; https://proksee.ca/) (27). Default parameters were used for all software unless otherwise specified.

**Fig 1:**
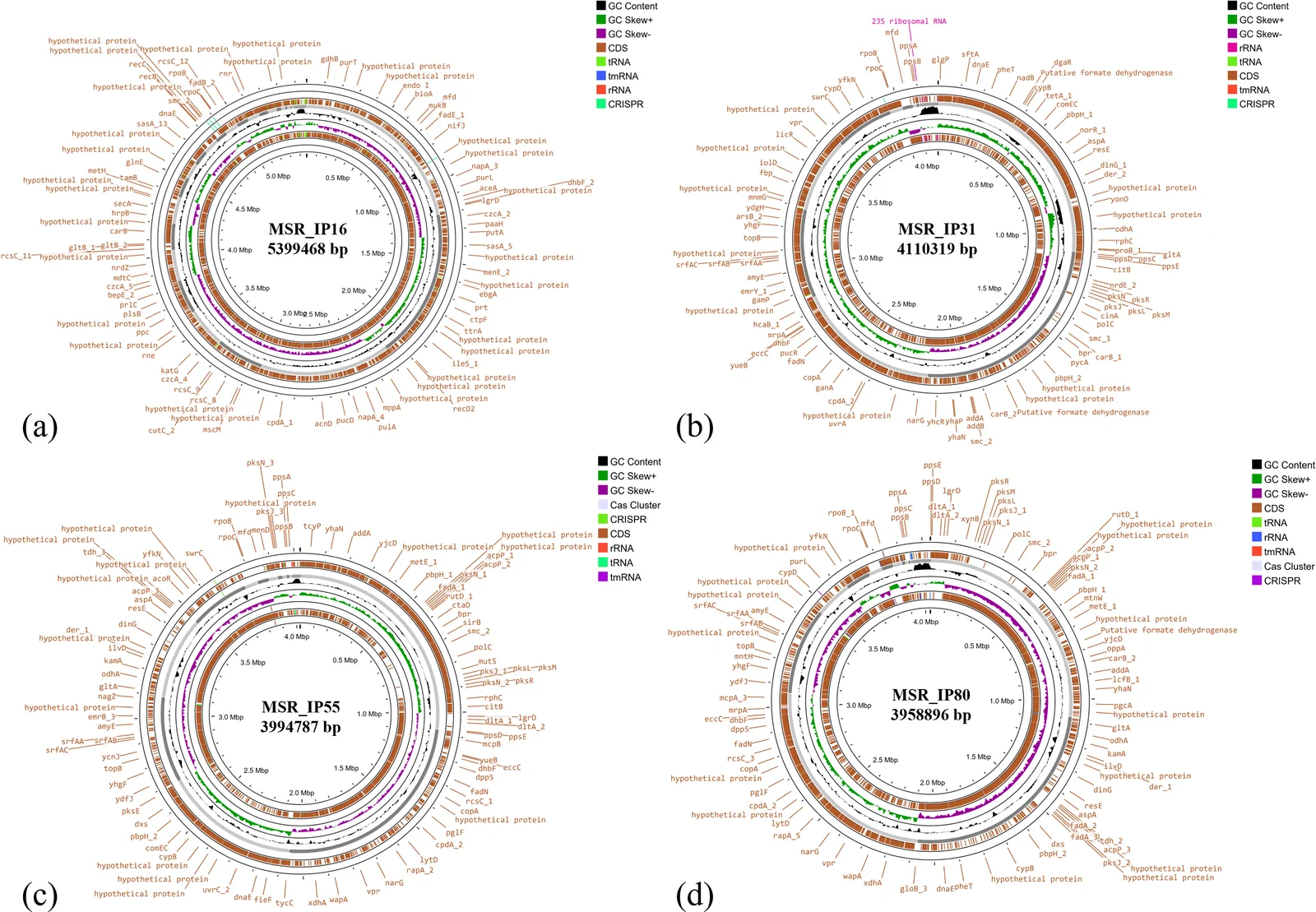
Circular genome representation of four potential indigenous probiotic strains isolated from sediment and shrimp samples. (**a**) *Photobacterium ganghwense* MSR_IP16, (**b**) *Bacillus subtilis* MSR_IP31, (**c**) *Bacillus velezensis* MSR_IP55, and (**d**) *Bacillus velezensis* MSR_IP80.

The strain information and genomic features of four potential indigenous probiotics were presented in Table 1. Genome annotation further demonstrated differences in coding and non-coding genetic elements among the strains (Fig. 1). Notably, CRISPR arrays were detected only in *P. ganghwense* MSR_IP16, suggesting the presence of adaptive immune defense mechanisms. In addition, no antibiotics resistance and plasmids were detected in all the four genomes. Collectively, genomic features along with biochemical tests, antagonistic, safety, and tolerance assays confirmed favorable profiles, establishing these strains as promising and safe probiotic candidates for sustainable shrimp aquaculture.

**TABLE 1:** Summary of the draft genome sequences of four potential indigenous probiotic strains isolated from sediment of Sundarbans mangrove region of Khulna and brood shrimps collected from the Bay of Bengal, Cox’s Bazar, Bangladesh

Variables	Strains data			
	MSR_IP16	MSR_IP31	MSR_IP55	MSR_IP80
Closest phylogenetic relatives in reference database	*Photobacterium ganghwense* JCM 12487	*Bacillus subtilis* 128	*Bacillus velezensis* NRRL B-41580	
ANI with type strain	98.70%	98.50%	97.80%	98.70%
Isolation source	Sediment		Brood shrimp	
Source location	Sundarbans mangrove		Bay of Bengal	
Geographic location	Khulna, Bangladesh		Cox’s Bazar, Bangladesh	
Geographical coordinates	22°16′35.40″N 89°24′07.36″E	22°17′20.39″N 89°23′29.12″E	21°11′08.81″N 91°52′15.01″E	
No. of paired-end reads (150 bp × 2)	66,710	67,244	77,121	73,922
Genome size (bp)	5,399,468	4,110,319	3,994,787	3,958,896
Contigs	44	21	19	26
Largest contig (bp)	1,201,170	1,106,134	1,079,983	1,080,237
Genome completeness (%)	94.46	99.41	99.41	99.41
Contamination (%)	4.27	0.52	0.05	0.19
Genome coverage (×)	35.94	48.33	59.54	54.95
GC content (%)	50.74	43.46	46.38	46.43
N50 (bp)	848,135	1,042,139	984,658	997,279
Genes	4,997	4,261	3,988	3,947
CDS	4,809	4,143	3,875	3,835
rRNAs	14	17	13	11
tRNAs	163	84	85	86
tmRNAs	1	1	1	1
Non-coding RNAs (ncRNAs)	23	30	20	20
ncRNA regions	31	66	60	60
CRISPR arrays	2	0	0	0
Small open reading frames	0	4	4	4
Origin of replication (oriCs)	1	2	2	3
Pseudogenes	10	16	14	14
BioSample accession	SAMN61029141	SAMN61029142	SAMN61029143	SAMN61029144
Sequence Read Archive (SRA)	SRR39247004	SRR39247003	SRR39247002	SRR39247001

## Data Availability

The genome sequences of *Photobacterium ganghwense* MSR_IP16, *Bacillus subtilis* MSR_IP31, *Bacillus velezensis* MSR_IP55, and *Bacillus velezensis* MSR_IP80 strains have been deposited in the NCBI GenBank database under the BioProject accession number PRJNA1480432. The BioSample accession numbers are SAMN61029141–SAMN61029144. The raw whole-genome sequencing data generated in this study have been deposited in the NCBI Sequence Read Archive (SRA) under accession numbers SRR39247001–SRR39247004.
